# Supplementary material for: blaNDM and mcr-1 to mcr-5 Gene Distribution Characteristics in Gut Specimens from Different Regions of China
Source: Antibiotics (Basel). 2021 Feb 25;10(3):233. doi: 10.3390/antibiotics10030233 (PMC7996585; doi:10.3390/antibiotics10030233)
Supplement: Supplementary file 1 [file antibiotics-10-00233-s001.zip › supplementary File/Table S1.docx]

**Table S1.** Background and genotypes of *mcr* or *bla*_NDM_ positive gut specimens

| ID | Source | Host | Specimen type | Region | Collection Year | Genotype |
| --- | --- | --- | --- | --- | --- | --- |
| 1 | livestock and poultry | canine | anal swab | Gansu | 2019 | *bla*_NDM-1_ |
| 2 | livestock and poultry | canine | anal swab | Gansu | 2019 | *bla*_NDM-1_ |
| 3 | livestock and poultry | canine | anal swab | Gansu | 2019 | *bla*_NDM-1_ |
| 4 | livestock and poultry | canine | anal swab | Gansu | 2019 | *bla*_NDM-1_ |
| 5 | diarrhea patient | human | feces | Guangxi | 2011 | *bla*_NDM-1_ |
| 6 | livestock and poultry | swine | feces | Anhui | 2019 | *bla*_NDM-1_ |
| 7 | livestock and poultry | swine | feces | Anhui | 2019 | *bla*_NDM-1_ |
| 8 | livestock and poultry | swine | feces | Anhui | 2019 | *bla*_NDM-1_ |
| 9 | poultry environment | chicken | depilator swab | Anhui | 2017 | *bla*_NDM-1_ |
| 10 | livestock and poultry | chicken | feces | Anhui | 2017 | *bla*_NDM-1_ |
| 11 | poultry environment | chicken | chopping board swab | Anhui | 2018 | *bla*_NDM-1_ |
| 12 | livestock and poultry | chicken | feces | Anhui | 2018 | *bla*_NDM-1_ |
| 13 | poultry environment | chicken | cage swab | Anhui | 2018 | *bla*_NDM-1_ |
| 14 | poultry environment | chicken | soil | Anhui | 2019 | *bla*_NDM-1_ |
| 15 | poultry environment | chicken | cage swab | Anhui | 2019 | *bla*_NDM-1_ |
| 16 | poultry environment | chicken | cage swab | Anhui | 2019 | *bla*_NDM-1_ |
| 17 | poultry environment | chicken | chopping board swab | Anhui | 2019 | *bla*_NDM-1_ |
| 18 | poultry environment | chicken | chopping board swab | Anhui | 2019 | *bla*_NDM-1_ |
| 19 | diarrhea patient | human | feces | Beijing | 2019 | *bla*_NDM-24_ |
| 20 | livestock and poultry | swine | oral-pharyngeal | Guangxi | 2014 | *mcr-1.1* |
| 21 | livestock and poultry | swine | oral-pharyngeal | Guangxi | 2014 | *mcr-1.1* |
| 22 | livestock and poultry | swine | oral-pharyngeal | Guangxi | 2014 | *mcr-1.1* |
| 23 | livestock and poultry | swine | oral-pharyngeal | Guangxi | 2014 | *mcr-1.1* |
| 24 | livestock and poultry | swine | oral-pharyngeal | Guangxi | 2014 | *mcr-1.1* |
| 25 | livestock and poultry | swine | oral-pharyngeal | Guangxi | 2014 | *mcr-1.1* |
| 26 | livestock and poultry | swine | oral-pharyngeal | Guangxi | 2014 | *mcr-1.1* |
| 27 | livestock and poultry | swine | oral-pharyngeal | Guangxi | 2014 | *mcr-1.1* |
| 28 | livestock and poultry | swine | oral-pharyngeal | Guangxi | 2014 | *mcr-1.1* |
| 29 | livestock and poultry | swine | oral-pharyngeal | Guangxi | 2014 | *mcr-1.1* |
| 30 | livestock and poultry | swine | oral-pharyngeal | Guangxi | 2014 | *mcr-1.1* |
| 31 | livestock and poultry | swine | oral-pharyngeal | Guangxi | 2014 | *mcr-1.1* |
| 32 | livestock and poultry | swine | feces | Guangxi | 2014 | *mcr-1.1* |
| 33 | livestock and poultry | swine | feces | Guangxi | 2014 | *mcr-1.1* |
| 34 | livestock and poultry | swine | feces | Guangxi | 2014 | *mcr-1.1* |
| 35 | livestock and poultry | swine | feces | Guangxi | 2014 | *mcr-1.1* |
| 36 | livestock and poultry | swine | feces | Guangxi | 2014 | *mcr-1.1* |
| 37 | livestock and poultry | swine | feces | Guangxi | 2014 | *mcr-1.1* |
| 38 | livestock and poultry | swine | feces | Guangxi | 2014 | *mcr-1.1* |
| 39 | livestock and poultry | swine | feces | Guangxi | 2014 | *mcr-1.1* |
| 40 | livestock and poultry | swine | feces | Guangxi | 2014 | *mcr-1.1* |
| 41 | livestock and poultry | swine | feces | Guangxi | 2014 | *mcr-1.1* |
| 42 | livestock and poultry | swine | feces | Guangxi | 2014 | *mcr-1.1* |
| 43 | livestock and poultry | swine | feces | Guangxi | 2014 | *mcr-1.1* |
| 44 | livestock and poultry | swine | feces | Guangxi | 2014 | *mcr-1.1* |
| 45 | livestock and poultry | swine | feces | Guangxi | 2014 | *mcr-1.1* |
| 46 | livestock and poultry | swine | feces | Guangxi | 2014 | *mcr-1.1* |
| 47 | livestock and poultry | swine | feces | Guangxi | 2014 | *mcr-1.1* |
| 48 | livestock and poultry | swine | feces | Guangxi | 2014 | *mcr-1.1* |
| 49 | livestock and poultry | swine | feces | Guangxi | 2014 | *mcr-1.1* |
| 50 | livestock and poultry | swine | feces | Guangxi | 2014 | *mcr-1.1* |
| 51 | livestock and poultry | swine | feces | Guangxi | 2014 | *mcr-1.1* |
| 52 | livestock and poultry | swine | feces | Guangxi | 2014 | *mcr-1.1* |
| 53 | livestock and poultry | swine | feces | Guangxi | 2014 | *mcr-1.1* |
| 54 | livestock and poultry | swine | feces | Guangxi | 2014 | *mcr-1.1* |
| 55 | livestock and poultry | swine | feces | Guangxi | 2014 | *mcr-1.1* |
| 56 | livestock and poultry | swine | feces | Guangxi | 2014 | *mcr-1.1* |
| 57 | livestock and poultry | swine | feces | Guangxi | 2014 | *mcr-1.1* |
| 58 | livestock and poultry | swine | feces | Guangxi | 2014 | *mcr-1.1* |
| 59 | livestock and poultry | swine | feces | Guangxi | 2014 | *mcr-1.1* |
| 60 | livestock and poultry | swine | feces | Guangxi | 2014 | *mcr-1.1* |
| 61 | livestock and poultry | swine | feces | Guangxi | 2014 | *mcr-1.1* |
| 62 | livestock and poultry | swine | feces | Guangxi | 2014 | *mcr-1.1* |
| 63 | livestock and poultry | swine | feces | Guangxi | 2014 | *mcr-1.1* |
| 64 | livestock and poultry | swine | feces | Guangxi | 2014 | *mcr-1.1* |
| 65 | livestock and poultry | swine | feces | Guangxi | 2014 | *mcr-1.1* |
| 66 | livestock and poultry | swine | feces | Guangxi | 2014 | *mcr-1.1* |
| 67 | livestock and poultry | swine | feces | Guangxi | 2014 | *mcr-1.1* |
| 68 | livestock and poultry | swine | feces | Guangxi | 2014 | *mcr-1.1* |
| 69 | livestock and poultry | swine | feces | Guangxi | 2014 | *mcr-1.1* |
| 70 | livestock and poultry | swine | feces | Guangxi | 2014 | *mcr-1.1* |
| 71 | livestock and poultry | swine | feces | Guangxi | 2014 | *mcr-1.1* |
| 72 | livestock and poultry | swine | feces | Guangxi | 2014 | *mcr-1.1* |
| 73 | livestock and poultry | swine | feces | Guangxi | 2014 | *mcr-1.1* |
| 74 | livestock and poultry | swine | feces | Guangxi | 2014 | *mcr-1.1* |
| 75 | livestock and poultry | swine | feces | Guangxi | 2014 | *mcr-1.1* |
| 76 | livestock and poultry | swine | feces | Guangxi | 2014 | *mcr-1.1* |
| 77 | livestock and poultry | swine | feces | Guangxi | 2014 | *mcr-1.1* |
| 78 | livestock and poultry | swine | feces | Guangxi | 2014 | *mcr-1.1* |
| 79 | livestock and poultry | swine | feces | Guangxi | 2014 | *mcr-1.1* |
| 80 | livestock and poultry | swine | feces | Guangxi | 2014 | *mcr-1.1* |
| 81 | livestock and poultry | swine | feces | Guangxi | 2014 | *mcr-1.1* |
| 82 | livestock and poultry | swine | feces | Guangxi | 2014 | *mcr-1.1* |
| 83 | livestock and poultry | swine | feces | Guangxi | 2014 | *mcr-1.1* |
| 84 | livestock and poultry | swine | feces | Guangxi | 2014 | *mcr-1.1* |
| 85 | livestock and poultry | swine | feces | Guangxi | 2014 | *mcr-1.1* |
| 86 | livestock and poultry | swine | feces | Guangxi | 2014 | *mcr-1.1* |
| 87 | livestock and poultry | swine | feces | Guangxi | 2014 | *mcr-1.1* |
| 88 | livestock and poultry | swine | feces | Guangxi | 2014 | *mcr-1.1* |
| 89 | livestock and poultry | swine | feces | Guangxi | 2014 | *mcr-1.1* |
| 90 | diarrhea patient | human | feces | Guangxi | 2011 | *mcr-1.1* |
| 91 | diarrhea patient | human | feces | Guangxi | 2011 | *mcr-1.1* |
| 92 | diarrhea patient | human | feces | Guangxi | 2011 | *mcr-1.1* |
| 93 | diarrhea patient | human | feces | Guangxi | 2011 | *mcr-1.1* |
| 94 | diarrhea patient | human | feces | Guangxi | 2011 | *mcr-1.1* |
| 95 | diarrhea patient | human | feces | Guangxi | 2011 | *mcr-1.1* |
| 96 | diarrhea patient | human | feces | Guangxi | 2011 | *mcr-1.1* |
| 97 | diarrhea patient | human | feces | Guangxi | 2011 | *mcr-1.1* |
| 98 | diarrhea patient | human | feces | Guangxi | 2011 | *mcr-1.1* |
| 99 | livestock and poultry | swine | feces | Anhui | 2019 | *mcr-1.1* |
| 100 | livestock and poultry | swine | feces | Anhui | 2019 | *mcr-1.1* |
| 101 | livestock and poultry | swine | feces | Anhui | 2019 | *mcr-1.1* |
| 102 | livestock and poultry | swine | feces | Anhui | 2019 | *mcr-1.1* |
| 103 | livestock and poultry | swine | feces | Anhui | 2019 | *mcr-1.1* |
| 104 | livestock and poultry | swine | feces | Anhui | 2019 | *mcr-1.1* |
| 105 | livestock and poultry | swine | feces | Anhui | 2019 | *mcr-1.1* |
| 106 | livestock and poultry | swine | feces | Anhui | 2019 | *mcr-1.1* |
| 107 | livestock and poultry | swine | feces | Anhui | 2019 | *mcr-1.1* |
| 108 | livestock and poultry | swine | feces | Anhui | 2019 | *mcr-1.1* |
| 109 | livestock and poultry | swine | feces | Anhui | 2019 | *mcr-1.1* |
| 110 | livestock and poultry | swine | feces | Anhui | 2019 | *mcr-1.1* |
| 111 | livestock and poultry | swine | feces | Anhui | 2019 | *mcr-1.1* |
| 112 | livestock and poultry | swine | feces | Anhui | 2019 | *mcr-1.1* |
| 113 | livestock and poultry | swine | feces | Anhui | 2019 | *mcr-1.1* |
| 114 | livestock and poultry | swine | feces | Anhui | 2019 | *mcr-1.1* |
| 115 | livestock and poultry | swine | feces | Anhui | 2019 | *mcr-1.1* |
| 116 | livestock and poultry | swine | feces | Anhui | 2019 | *mcr-1.1* |
| 117 | livestock and poultry | swine | feces | Anhui | 2019 | *mcr-1.1* |
| 118 | livestock and poultry | swine | feces | Anhui | 2019 | *mcr-1.1* |
| 119 | livestock and poultry | swine | feces | Anhui | 2019 | *mcr-1.1* |
| 120 | livestock and poultry | swine | feces | Anhui | 2019 | *mcr-1.1* |
| 121 | livestock and poultry | swine | feces | Anhui | 2019 | *mcr-1.1* |
| 122 | livestock and poultry | swine | feces | Anhui | 2019 | *mcr-1.1* |
| 123 | livestock and poultry | swine | feces | Anhui | 2019 | *mcr-1.1* |
| 124 | livestock and poultry | swine | feces | Anhui | 2019 | *mcr-1.1* |
| 125 | livestock and poultry | swine | feces | Anhui | 2019 | *mcr-1.1* |
| 126 | livestock and poultry | swine | feces | Anhui | 2019 | *mcr-1.1* |
| 127 | livestock and poultry | swine | feces | Anhui | 2019 | *mcr-1.1* |
| 128 | livestock and poultry | swine | feces | Anhui | 2019 | *mcr-1.1* |
| 129 | livestock and poultry | swine | feces | Anhui | 2019 | *mcr-1.1* |
| 130 | livestock and poultry | swine | feces | Anhui | 2019 | *mcr-1.1* |
| 131 | livestock and poultry | swine | feces | Anhui | 2019 | *mcr-1.1* |
| 132 | livestock and poultry | swine | feces | Anhui | 2019 | *mcr-1.1* |
| 133 | livestock and poultry | swine | feces | Anhui | 2019 | *mcr-1.1* |
| 134 | livestock and poultry | swine | feces | Anhui | 2019 | *mcr-1.1* |
| 135 | livestock and poultry | swine | feces | Anhui | 2019 | *mcr-1.1* |
| 136 | livestock and poultry | swine | feces | Anhui | 2019 | *mcr-1.1* |
| 137 | livestock and poultry | swine | feces | Anhui | 2019 | *mcr-1.1* |
| 138 | livestock and poultry | swine | feces | Anhui | 2019 | *mcr-1.1* |
| 139 | livestock and poultry | swine | feces | Anhui | 2019 | *mcr-1.1* |
| 140 | livestock and poultry | swine | feces | Anhui | 2019 | *mcr-1.1* |
| 141 | livestock and poultry | swine | feces | Anhui | 2019 | *mcr-1.1* |
| 142 | livestock and poultry | swine | feces | Anhui | 2019 | *mcr-1.1* |
| 143 | livestock and poultry | swine | feces | Anhui | 2019 | *mcr-1.1* |
| 144 | livestock and poultry | swine | feces | Anhui | 2019 | *mcr-1.1* |
| 145 | livestock and poultry | swine | feces | Anhui | 2019 | *mcr-1.1* |
| 146 | livestock and poultry | swine | feces | Anhui | 2019 | *mcr-1.1* |
| 147 | livestock and poultry | swine | feces | Anhui | 2019 | *mcr-1.1* |
| 148 | livestock and poultry | swine | feces | Anhui | 2019 | *mcr-1.1* |
| 149 | livestock and poultry | swine | feces | Anhui | 2019 | *mcr-1.1* |
| 150 | livestock and poultry | swine | feces | Anhui | 2019 | *mcr-1.1* |
| 151 | livestock and poultry | swine | feces | Anhui | 2019 | *mcr-1.1* |
| 152 | livestock and poultry | swine | feces | Anhui | 2019 | *mcr-1.1* |
| 153 | livestock and poultry | swine | feces | Anhui | 2019 | *mcr-1.1* |
| 154 | livestock and poultry | swine | feces | Anhui | 2019 | *mcr-1.1* |
| 155 | livestock and poultry | swine | feces | Anhui | 2019 | *mcr-1.1* |
| 156 | livestock and poultry | swine | feces | Anhui | 2019 | *mcr-1.1* |
| 157 | livestock and poultry | swine | feces | Anhui | 2019 | *mcr-1.1* |
| 158 | livestock and poultry | swine | feces | Anhui | 2019 | *mcr-1.1* |
| 159 | livestock and poultry | swine | feces | Anhui | 2019 | *mcr-1.1* |
| 160 | livestock and poultry | swine | feces | Anhui | 2019 | *mcr-1.1* |
| 161 | livestock and poultry | swine | feces | Anhui | 2019 | *mcr-1.1* |
| 162 | wild animal | bat | intestinal content | Guangxi | 2012 | *mcr-1.1* |
| 163 | wild animal | bat | intestinal content | Guangxi | 2012 | *mcr-1.1* |
| 164 | wild animal | bat | intestinal content | Guangxi | 2012 | *mcr-1.1* |
| 165 | wild animal | bat | intestinal content | Guangxi | 2012 | *mcr-1.1* |
| 166 | wild animal | bat | intestinal content | Guangxi | 2012 | *mcr-1.1* |
| 167 | wild animal | bat | oral-pharyngeal | Guangxi | 2012 | *mcr-1.1* |
| 168 | wild animal | bat | oral-pharyngeal | Guangxi | 2012 | *mcr-1.1* |
| 169 | wild animal | bat | oral-pharyngeal | Guangxi | 2012 | *mcr-1.1* |
| 170 | livestock and poultry | canine | anal swab | Gansu | 2019 | *mcr-1.1* |
| 171 | livestock and poultry | canine | anal swab | Gansu | 2019 | *mcr-1.1* |
| 172 | livestock and poultry | canine | anal swab | Gansu | 2019 | *mcr-1.1* |
| 173 | livestock and poultry | canine | anal swab | Gansu | 2019 | *mcr-1.1* |
| 174 | livestock and poultry | canine | anal swab | Gansu | 2019 | *mcr-1.1* |
| 175 | livestock and poultry | canine | anal swab | Gansu | 2019 | *mcr-1.1* |
| 176 | livestock and poultry | canine | anal swab | Gansu | 2019 | *mcr-1.1* |
| 177 | livestock and poultry | canine | anal swab | Gansu | 2019 | *mcr-1.1* |
| 178 | livestock and poultry | canine | anal swab | Gansu | 2019 | *mcr-1.1* |
| 179 | livestock and poultry | canine | anal swab | Gansu | 2019 | *mcr-1.1* |
| 180 | livestock and poultry | canine | anal swab | Gansu | 2019 | *mcr-1.1* |
| 181 | livestock and poultry | canine | anal swab | Gansu | 2019 | *mcr-1.1* |
| 182 | livestock and poultry | canine | anal swab | Gansu | 2019 | *mcr-1.1* |
| 183 | livestock and poultry | chicken | feces | Anhui | 2017 | *mcr-1.1* |
| 184 | livestock and poultry | chicken | feces | Anhui | 2017 | *mcr-1.1* |
| 185 | poultry environment | chicken | cage swab | Anhui | 2018 | *mcr-1.1* |
| 186 | livestock and poultry | chicken | feces | Anhui | 2018 | *mcr-1.1* |
| 187 | poultry environment | chicken | chopping board swab | Anhui | 2018 | *mcr-1.1* |
| 188 | poultry environment | chicken | chopping board swab | Anhui | 2018 | *mcr-1.1* |
| 189 | livestock and poultry | chicken | feces | Anhui | 2018 | *mcr-1.1* |
| 190 | poultry environment | poultry | cleaning sewage | Anhui | 2018 | *mcr-1.1* |
| 191 | livestock and poultry | chicken | feces | Anhui | 2018 | *mcr-1.1* |
| 192 | livestock and poultry | chicken | feces | Anhui | 2018 | *mcr-1.1* |
| 193 | poultry environment | chicken | cage swab | Anhui | 2018 | *mcr-1.1* |
| 194 | livestock and poultry | chicken | feces | Anhui | 2018 | *mcr-1.1* |
| 195 | livestock and poultry | chicken | feces | Anhui | 2018 | *mcr-1.1* |
| 196 | poultry environment | chicken | cage swab | Anhui | 2018 | *mcr-1.1* |
| 197 | poultry environment | chicken | chopping board swab | Anhui | 2018 | *mcr-1.1* |
| 198 | poultry environment | poultry | cleaning sewage | Anhui | 2018 | *mcr-1.1* |
| 199 | poultry environment | chicken | depilator swab | Anhui | 2018 | *mcr-1.1* |
| 200 | poultry environment | chicken | cage swab | Anhui | 2018 | *mcr-1.1* |
| 201 | livestock and poultry | chicken | feces | Anhui | 2018 | *mcr-1.1* |
| 202 | poultry environment | chicken | depilator swab | Anhui | 2018 | *mcr-1.1* |
| 203 | livestock and poultry | chicken | feces | Anhui | 2018 | *mcr-1.1* |
| 204 | livestock and poultry | chicken | feces | Anhui | 2018 | *mcr-1.1* |
| 205 | livestock and poultry | chicken | feces | Anhui | 2019 | *mcr-1.1* |
| 206 | livestock and poultry | chicken | feces | Anhui | 2019 | *mcr-1.1* |
| 207 | livestock and poultry | chicken | feces | Anhui | 2019 | *mcr-1.1* |
| 208 | poultry environment | chicken | soil | Anhui | 2019 | *mcr-1.1* |
| 209 | livestock and poultry | chicken | feces | Anhui | 2019 | *mcr-1.1* |
| 210 | livestock and poultry | chicken | feces | Anhui | 2019 | *mcr-1.1* |
| 211 | livestock and poultry | chicken | feces | Anhui | 2019 | *mcr-1.1* |
| 212 | livestock and poultry | chicken | feces | Anhui | 2019 | *mcr-1.1* |
| 213 | livestock and poultry | chicken | feces | Anhui | 2019 | *mcr-1.1* |
| 214 | livestock and poultry | chicken | feces | Anhui | 2019 | *mcr-1.1* |
| 215 | livestock and poultry | chicken | feces | Anhui | 2019 | *mcr-1.1* |
| 216 | poultry environment | chicken | soil | Anhui | 2019 | *mcr-1.1* |
| 217 | livestock and poultry | chicken | feces | Anhui | 2019 | *mcr-1.1* |
| 218 | livestock and poultry | chicken | feces | Anhui | 2019 | *mcr-1.1* |
| 219 | livestock and poultry | chicken | feces | Anhui | 2019 | *mcr-1.1* |
| 220 | livestock and poultry | chicken | feces | Anhui | 2019 | *mcr-1.1* |
| 221 | poultry environment | chicken | cage swab | Anhui | 2019 | *mcr-1.1* |
| 222 | poultry environment | chicken | chopping board swab | Anhui | 2019 | *mcr-1.1* |
| 223 | poultry environment | chicken | chopping board swab | Anhui | 2019 | *mcr-1.1* |
| 224 | poultry environment | chicken | chopping board swab | Anhui | 2019 | *mcr-1.1* |
| 225 | livestock and poultry | chicken | feces | Anhui | 2019 | *mcr-1.1* |
| 226 | livestock and poultry | chicken | feces | Anhui | 2019 | *mcr-1.1* |
| 227 | livestock and poultry | chicken | feces | Anhui | 2019 | *mcr-1.1* |
| 228 | livestock and poultry | chicken | feces | Anhui | 2019 | *mcr-1.1* |
| 229 | livestock and poultry | chicken | feces | Anhui | 2019 | *mcr-1.1* |
| 230 | livestock and poultry | chicken | feces | Anhui | 2019 | *mcr-1.1* |
| 231 | livestock and poultry | chicken | feces | Anhui | 2019 | *mcr-1.1* |
| 232 | livestock and poultry | chicken | feces | Anhui | 2019 | *mcr-1.1* |
| 233 | livestock and poultry | chicken | feces | Anhui | 2019 | *mcr-1.1* |
| 234 | livestock and poultry | chicken | feces | Anhui | 2019 | *mcr-1.1* |
| 235 | livestock and poultry | chicken | feces | Anhui | 2019 | *mcr-1.1* |
| 236 | livestock and poultry | chicken | feces | Anhui | 2019 | *mcr-1.1* |
| 237 | livestock and poultry | chicken | feces | Anhui | 2019 | *mcr-1.1* |
| 238 | livestock and poultry | chicken | feces | Anhui | 2019 | *mcr-1.1* |
| 239 | poultry environment | chicken | cage swab | Anhui | 2018 | *mcr-1.1* |
| 240 | livestock and poultry | chicken | feces | Anhui | 2018 | *mcr-1.1* |
| 241 | livestock and poultry | chicken | feces | Anhui | 2018 | *mcr-1.1* |
| 242 | poultry environment | poultry | cleaning sewage | Anhui | 2018 | *mcr-1.1* |
| 243 | livestock and poultry | chicken | feces | Anhui | 2018 | *mcr-1.1* |
| 244 | livestock and poultry | chicken | feces | Anhui | 2018 | *mcr-1.1* |
| 245 | livestock and poultry | chicken | feces | Anhui | 2019 | *mcr-1.1* |
| 246 | poultry environment | chicken | cage swab | Anhui | 2019 | *mcr-1.1* |
| 247 | poultry environment | chicken | chopping board swab | Anhui | 2019 | *mcr-1.1* |
| 248 | poultry environment | chicken | depilator swab | Anhui | 2019 | *mcr-1.1* |
| 249 | poultry environment | poultry | cleaning sewage | Anhui | 2019 | *mcr-1.1* |
| 250 | poultry environment | chicken | chopping board swab | Anhui | 2019 | *mcr-1.1* |
| 251 | livestock and poultry | chicken | feces | Anhui | 2019 | *mcr-1.1* |
| 252 | poultry environment | chicken | chopping board swab | Anhui | 2019 | *mcr-1.1* |
| 253 | poultry environment | chicken | depilator swab | Anhui | 2019 | *mcr-1.1* |
| 254 | poultry environment | poultry | cleaning sewage | Anhui | 2019 | *mcr-1.1* |
| 255 | livestock and poultry | chicken | feces | Anhui | 2019 | *mcr-1.1* |
| 256 | poultry environment | chicken | drinking water | Anhui | 2019 | *mcr-1.1* |
| 257 | livestock and poultry | chicken | feces | Anhui | 2019 | *mcr-1.1* |
| 258 | livestock and poultry | chicken | feces | Anhui | 2019 | *mcr-1.1* |
| 259 | poultry environment | chicken | cage swab | Anhui | 2019 | *mcr-1.1* |
| 260 | poultry environment | chicken | drinking water | Anhui | 2019 | *mcr-1.1* |
| 261 | poultry environment | chicken | chopping board swab | Anhui | 2019 | *mcr-1.1* |
| 262 | poultry environment | poultry | cleaning sewage | Anhui | 2019 | *mcr-1.1* |
| 263 | livestock and poultry | chicken | feces | Anhui | 2019 | *mcr-1.1* |
| 264 | livestock and poultry | chicken | feces | Anhui | 2019 | *mcr-1.1* |
| 265 | livestock and poultry | chicken | feces | Anhui | 2019 | *mcr-1.1* |
| 266 | livestock and poultry | chicken | feces | Anhui | 2019 | *mcr-1.1* |
| 267 | livestock and poultry | chicken | feces | Anhui | 2019 | *mcr-1.1* |
| 268 | poultry environment | chicken | cage swab | Anhui | 2019 | *mcr-1.1* |
| 269 | poultry environment | poultry | cleaning sewage | Anhui | 2019 | *mcr-1.1* |
| 270 | livestock and poultry | chicken | feces | Anhui | 2019 | *mcr-1.1* |
| 271 | poultry environment | poultry | cleaning sewage | Anhui | 2019 | *mcr-1.1* |
| 272 | livestock and poultry | chicken | feces | Anhui | 2019 | *mcr-1.1* |
| 273 | poultry environment | poultry | cleaning sewage | Anhui | 2019 | *mcr-1.1* |
| 274 | poultry environment | poultry | cleaning sewage | Anhui | 2019 | *mcr-1.1* |
| 275 | livestock and poultry | chicken | feces | Anhui | 2019 | *mcr-1.1* |
| 276 | poultry environment | poultry | cleaning sewage | Anhui | 2019 | *mcr-1.1* |
| 277 | poultry environment | poultry | cleaning sewage | Anhui | 2019 | *mcr-1.1* |
| 278 | poultry environment | poultry | cleaning sewage | Anhui | 2019 | *mcr-1.1* |
| 279 | poultry environment | poultry | cleaning sewage | Anhui | 2019 | *mcr-1.1* |
| 280 | poultry environment | chicken | chopping board swab | Anhui | 2019 | *mcr-1.1* |
| 281 | poultry environment | poultry | cleaning sewage | Anhui | 2019 | *mcr-1.1* |
| 282 | poultry environment | poultry | cleaning sewage | Anhui | 2019 | *mcr-1.1* |
| 283 | poultry environment | chicken | drinking water | Anhui | 2019 | *mcr-1.1* |
| 284 | poultry environment | chicken | drinking water | Anhui | 2019 | *mcr-1.1* |
| 285 | poultry environment | poultry | cleaning sewage | Anhui | 2019 | *mcr-1.1* |
| 286 | poultry environment | chicken | chopping board swab | Anhui | 2019 | *mcr-1.1* |
| 287 | livestock and poultry | swine | oral-pharyngeal | Anhui | 2015 | *mcr-1.1* |
| 288 | livestock and poultry | swine | oral-pharyngeal | Yunnan | 2010 | *mcr-1.1* |
| 289 | livestock and poultry | swine | oral-pharyngeal | Yunnan | 2010 | *mcr-1.1* |
| 290 | diarrhea patient | human | feces | Yunnan | 2010 | *mcr-1.1* |
| 291 | livestock and poultry | swine | feces | Yunnan | 2013 | *mcr-1.1* |
| 292 | livestock and poultry | swine | feces | Yunnan | 2013 | *mcr-1.1* |
| 293 | livestock and poultry | swine | feces | Yunnan | 2013 | *mcr-1.1* |
| 294 | livestock and poultry | swine | feces | Yunnan | 2013 | *mcr-1.1* |
| 295 | livestock and poultry | swine | feces | Yunnan | 2013 | *mcr-1.1* |
| 296 | livestock and poultry | swine | feces | Yunnan | 2013 | *mcr-1.1* |
| 297 | livestock and poultry | swine | feces | Yunnan | 2013 | *mcr-1.1* |
| 298 | livestock and poultry | swine | feces | Yunnan | 2013 | *mcr-1.1* |
| 299 | livestock and poultry | swine | feces | Yunnan | 2013 | *mcr-1.1* |
| 300 | livestock and poultry | swine | feces | Yunnan | 2013 | *mcr-1.1* |
| 301 | livestock and poultry | swine | feces | Yunnan | 2013 | *mcr-1.1* |
| 302 | livestock and poultry | swine | feces | Yunnan | 2013 | *mcr-1.1* |
| 303 | livestock and poultry | swine | feces | Yunnan | 2013 | *mcr-1.1* |
| 304 | livestock and poultry | swine | feces | Qinghai | 2011 | *mcr-1.1* |
| 305 | livestock and poultry | swine | feces | Qinghai | 2011 | *mcr-1.1* |
| 306 | livestock and poultry | swine | feces | Qinghai | 2011 | *mcr-1.1* |
| 307 | livestock and poultry | swine | feces | Qinghai | 2011 | *mcr-1.1* |
| 308 | livestock and poultry | swine | feces | Qinghai | 2011 | *mcr-1.1* |
| 309 | livestock and poultry | swine | feces | Qinghai | 2011 | *mcr-1.1* |
| 310 | livestock and poultry | swine | feces | Qinghai | 2011 | *mcr-1.1* |
| 311 | livestock and poultry | swine | feces | Qinghai | 2011 | *mcr-1.1* |
| 312 | livestock and poultry | swine | feces | Qinghai | 2011 | *mcr-1.1* |
| 313 | livestock and poultry | swine | feces | Qinghai | 2011 | *mcr-1.1* |
| 314 | diarrhea patient | human | feces | Beijing | 2013 | *mcr-1.1* |
| 315 | diarrhea patient | human | feces | Beijing | 2013 | *mcr-1.1* |
| 316 | diarrhea patient | human | feces | Beijing | 2013 | *mcr-1.1* |
| 317 | diarrhea patient | human | feces | Beijing | 2013 | *mcr-1.1* |
| 318 | diarrhea patient | human | feces | Beijing | 2014 | *mcr-1.1* |
| 319 | diarrhea patient | human | feces | Beijing | 2014 | *mcr-1.1* |
| 320 | diarrhea patient | human | feces | Beijing | 2015 | *mcr-1.1* |
| 321 | wild animal | rat | intestinal content | Guizhou | 2015 | *mcr-1.1* |
| 322 | wild animal | marmot | oral-pharyngeal | Gansu | 2016 | *mcr-1.1* |
| 323 | wild animal | marmot | oral-pharyngeal | Gansu | 2016 | *mcr-1.1* |
| 324 | wild animal | marmot | oral-pharyngeal | Gansu | 2017 | *mcr-1.1* |
| 325 | poultry environment | chicken | cage swab | Anhui | 2018 | *mcr-1.29* |
| 326 | livestock and poultry | swine | oral-pharyngeal | Guangxi | 2014 | *mcr-1.30* |
| 327 | livestock and poultry | swine | oral-pharyngeal | Guangxi | 2014 | *mcr-2.4* |
| 328 | livestock and poultry | swine | oral-pharyngeal | Guangxi | 2014 | *mcr-2.4* |
| 329 | livestock and poultry | swine | oral-pharyngeal | Guangxi | 2014 | *mcr-2.4* |
| 330 | livestock and poultry | swine | oral-pharyngeal | Guangxi | 2014 | *mcr-2.6* |
| 331 | poultry environment | poultry | cleaning sewage | Anhui | 2019 | *mcr-3.18* |
| 332 | poultry environment | chicken | drinking water | Anhui | 2019 | *mcr-3.18* |
| 333 | poultry environment | poultry | cleaning sewage | Anhui | 2019 | *mcr-3.3* |
| 334 | wild animal | bat | oral-pharyngeal | Guangxi | 2012 | *mcr-3.31* |
| 335 | livestock and poultry | swine | oral-pharyngeal | Yunnan | 2010 | *mcr-3.32* |
| 336 | livestock and poultry | swine | oral-pharyngeal | Yunnan | 2010 | *mcr-3.32* |
| 337 | livestock and poultry | swine | oral-pharyngeal | Yunnan | 2010 | *mcr-3.32* |
| 338 | livestock and poultry | swine | oral-pharyngeal | Yunnan | 2010 | *mcr-3.32* |
| 339 | livestock and poultry | swine | oral-pharyngeal | Yunnan | 2010 | *mcr-3.32* |
| 340 | livestock and poultry | swine | oral-pharyngeal | Yunnan | 2010 | *mcr-3.32* |
| 341 | livestock and poultry | swine | feces | Guangxi | 2014 | *mcr-4.3* |
| 342 | livestock and poultry | fish | intestinal swab | Anhui | 2019 | *mcr-4.3* |
| 343 | livestock and poultry | fish | intestinal swab | Anhui | 2019 | *mcr-4.3* |
| 344 | livestock and poultry | fish | intestinal swab | Anhui | 2019 | *mcr-4.3* |
| 345 | livestock and poultry | swine | feces | Qinghai | 2011 | *mcr-4.3* |
| 346 | livestock and poultry | swine | feces | Anhui | 2015 | *mcr-4.3* |
| 347 | livestock and poultry | swine | feces | Anhui | 2015 | *mcr-4.3* |
| 348 | livestock and poultry | swine | feces | Anhui | 2019 | *bla*_NDM-1_, *mcr-1.1* |
| 349 | livestock and poultry | swine | feces | Anhui | 2019 | *bla*_NDM-1_, *mcr-1.1* |
| 350 | livestock and poultry | swine | feces | Anhui | 2019 | *bla*_NDM-1_, *mcr-1.1* |
| 351 | livestock and poultry | chicken | feces | Anhui | 2018 | *bla*_NDM-1_, *mcr-1.1* |
| 352 | livestock and poultry | chicken | feces | Anhui | 2018 | *bla*_NDM-1_, *mcr-1.1* |
| 353 | poultry environment | poultry | cleaning sewage | Anhui | 2019 | *bla*_NDM-1_, *mcr-1.1* |
| 354 | poultry environment | chicken | chopping board swab | Anhui | 2019 | *bla*_NDM-1_, *mcr-1.1* |
| 355 | livestock and poultry | chicken | feces | Anhui | 2019 | *bla*_NDM-1_, *mcr-1.1* |
| 356 | poultry environment | chicken | cage swab | Anhui | 2019 | *bla*_NDM-24_, *mcr-1.1* |
| 357 | livestock and poultry | chicken | feces | Anhui | 2019 | *bla*_NDM-24_, *mcr-1.1* |
| 358 | livestock and poultry | swine | oral-pharyngeal | Guangxi | 2014 | *mcr-1.1*, *mcr-2.4* |
| 359 | livestock and poultry | swine | oral-pharyngeal | Guangxi | 2014 | *mcr-1.1*, *mcr-2.4* |
| 360 | livestock and poultry | swine | oral-pharyngeal | Guangxi | 2014 | *mcr-1.1*, *mcr-2.5* |
| 361 | livestock and poultry | swine | oral-pharyngeal | Guangxi | 2014 | *mcr-1.1*, *mcr-2.7* |
| 362 | livestock and poultry | swine | oral-pharyngeal | Guangxi | 2014 | *mcr-1.1*, *mcr-2.7* |
| 363 | livestock and poultry | swine | oral-pharyngeal | Guangxi | 2014 | *mcr-1.1*, *mcr-2.7* |
| 364 | livestock and poultry | swine | oral-pharyngeal | Guangxi | 2014 | *mcr-1.1*, *mcr-2.7* |
| 365 | livestock and poultry | swine | oral-pharyngeal | Guangxi | 2014 | *mcr-1.1*, *mcr-2.7* |
| 366 | livestock and poultry | swine | oral-pharyngeal | Guangxi | 2014 | *mcr-1.1*, *mcr-2.7* |
| 367 | livestock and poultry | swine | oral-pharyngeal | Guangxi | 2014 | *mcr-1.1*, *mcr-2.7* |
| 368 | livestock and poultry | swine | oral-pharyngeal | Guangxi | 2014 | *mcr-1.1*, *mcr-2.7* |
| 369 | livestock and poultry | swine | oral-pharyngeal | Guangxi | 2014 | *mcr-1.1*, *mcr-2.7* |
| 370 | poultry environment | poultry | cleaning sewage | Anhui | 2019 | *mcr-1.1*, *mcr-3.3* |
| 371 | livestock and poultry | swine | feces | Guangxi | 2014 | *mcr-1.1*, *mcr-4.3* |
| 372 | livestock and poultry | swine | oral-pharyngeal | Guangxi | 2014 | *mcr-1.30*, *mcr-2.7* |
| 373 | livestock and poultry | swine | oral-pharyngeal | Guangxi | 2014 | *mcr-1.30*, *mcr-2.7* |
| 374 | livestock and poultry | swine | oral-pharyngeal | Guangxi | 2014 | *mcr-1.30*, *mcr-2.7* |
| 375 | livestock and poultry | swine | oral-pharyngeal | Guangxi | 2014 | *mcr-1.30*, *mcr-2.7* |
