# Supplementary material for: blaNDM and mcr-1 to mcr-5 Gene Distribution Characteristics in Gut Specimens from Different Regions of China
Source: Antibiotics (Basel). 2021 Feb 25;10(3):233. doi: 10.3390/antibiotics10030233 (PMC7996585; doi:10.3390/antibiotics10030233)
Supplement: Supplementary file 1 [file antibiotics-10-00233-s001.zip › supplementary File/Table S2 .docx]

**Table S2.** Primers for *bla*_NDM_ and *mcr* screening and CDS amplification.

| **Target gene** | **primer** | **Sequence (5'→3')** | **Product size (bp)** | **Annealing temperature** | **Reference** |
| --- | --- | --- | --- | --- | --- |
| *bla*_NDM_ | NDM-1_17U-F | CAGCACACTTCCTATCTC | 291bp^a^ | 54℃ | [20] |
|  | NDM-1_17U-R | CCGCAACCATCCCCTCTT |  |  |  |
| *mcr-1* | MCR-1_CLR5-F | CGGTCAGTCCGTTTGTTC | 309bp^a^ | 54℃ |  |
|  | MCR-1_CLR5-R | CTTGGTCGGTCTGTAGGG |  |  |  |
| *mcr-1* | MCR-1-/2-Fw | TATCGCTATGTGCTAAAGCCTG | 1139bp^a^ | 56℃ | [41] |
|  | MCR-1-Rv | CGTCTGCAGCCACTGG |  |  |  |
| *mcr-2* | MCR-1-/2-Fw | TATCGCTATGTGCTAAAGCCTG | 816bp^a^ | 56℃ |  |
|  | MCR-2-Rv | AAAATACTGCGTGGCAGGTAGC |  |  |  |
| *mcr-3* | MCR-3-Fw | CAATCGTTAGTTACACAATGATGAAG | 676bp^a^ | 56℃ |  |
|  | MCR-3-Rv | AACACATCTAGCAGGCCCTC |  |  |  |
| *mcr-4* | MCR-4-Fw | ATCCTGCTGAAGCATTGATG | 405bp^a^ | 56℃ |  |
|  | MCR-4-Rv | GCGCGCAGTTTCACC |  |  |  |
| *mcr-5* | MCR-5-Fw | GGTTGAGCGGCTATGAAC | 207bp^a^ | 56℃ |  |
|  | MCR-5-Rv | GAATGTTGACGTCACTACGG |  |  |  |
| *mcr-1* | mcr-1 FL-F | AGAAGCACTGGGTGTAGAAT | 2189bp^b^ | 54℃ | [20] |
|  | mcr-1 FL-R | GCCATGACAAGAGCGATA |  |  |  |
| *mcr-1* | FR-mcr-FL-F | CATCAATCAGTGGAGCG | 2060bp^b^ | 54℃ |  |
|  | FR-mcr-FL-R | CTCATCTCAGCAAGTAGG |  |  |  |
| *mcr-1* | DR-mcr-FL-F | GCAGTATAATTGCCGTAA | 1841bp^b^ | 50℃ |  |
|  | DR-mcr-FL-R | CTGACTGTGCTCAAGGGT |  |  |  |
| *mcr-2* | mcr2L-2F | ACGACATTGAAGTATAATCGCCAAC | 1721bp^b^ | 57℃ | this study |
|  | mcr2L-2R | CACGATACCGTAGTGTGATGGCT |  |  |  |
| *mcr-2* | mcr2L-2FS | ACGACATTGAAGTATAATCGCC | 1718bp^b^ | 52℃ |  |
|  | mcr2L-2RS | GATACCGTAGTGTGATGGCT |  |  |  |
| *mcr-3* | mcr3L-8F2 | CCAAAGCCAATAGCAGTA | 2010bp^b^ | 47℃ |  |
|  | mcr3L-8R2 | TTCGGATAAGACATTTGTGA |  |  |  |

a, Screening; b, CDS amplification
